# A Comprehensive Review of Detection Methods for SARS-CoV-2

**DOI:** 10.3390/microorganisms9020232

**Published:** 2021-01-22

**Authors:** Aziz Eftekhari, Mahdieh Alipour, Leila Chodari, Solmaz Maleki Dizaj, Mohammadreza Ardalan, Mohammad Samiei, Simin Sharifi, Sepideh Zununi Vahed, Irada Huseynova, Rovshan Khalilov, Elham Ahmadian, Magali Cucchiarini

**Affiliations:** 1 Pharmacology and Toxicology Department, Maragheh University of Medical Sciences, Maragheh 5515878151, Iran; eftekharia@tbzmed.ac.ir; 2 Dental and Periodontal Research Center, Faculty of Dentistry, Tabriz University of Medical Sciences, Tabriz 5166615731, Iran; alipour.m@tbzmed.ac.ir (M.A.); maleki.s.89@gmail.com (S.M.D.); sharifi.simin7@gmail.com (S.S.); 3 Physiology Department, Faculty of Medicine, Urmia University of Medical Sciences, Urmia 571478334, Iran; lchodari@yahoo.com; 4 Kidney Research Center, Imam Reza Hospital, Tabriz University of Medical Sciences, Tabriz 5166615731, Iran; ardalanm34@gmail.com (M.A.); sepide.zununi@gmail.com (S.Z.V.); 5 Faculty of Dentistry, Tabriz University of Medical Sciences, Tabriz 5166615731, Iran; samiei.moh@gmail.com; 6 Institute of Molecular Biology & Biotechnologies, Azerbaijan National Academy of Sciences, 11 Izzat Nabiyev, Baku AZ 1073, Azerbaijan; i_guseinova@mail.ru; 7 Department of Biophysics and Biochemistry, Baku State University, Baku AZ 1148, Azerbaijan; hrovshan@hotmail.com; 8 Joint Ukraine-Azerbaijan International Research and Education Center of Nanobiotechnology and Functional Nanosystems, 82100 Drohobych, Ukraine; 9 Center of Experimental Orthopaedics, Saarland University Medical Center, D-66421 Homburg/Saar, Germany

**Keywords:** COVID-19, coronavirus, detection, epidemic, nanotechnology

## Abstract

Recently, the outbreak of the coronavirus disease 2019 (COVID-19), caused by the SARS-CoV-2 virus, in China and its subsequent spread across the world has caused numerous infections and deaths and disrupted normal social activity. Presently, various techniques are used for the diagnosis of SARS-CoV-2 infection, with various advantages and weaknesses to each. In this paper, we summarize promising methods, such as reverse transcription-polymerase chain reaction (RT-PCR), serological testing, point-of-care testing, smartphone surveillance of infectious diseases, nanotechnology-based approaches, biosensors, amplicon-based metagenomic sequencing, smartphone, and wastewater-based epidemiology (WBE) that can also be utilized for the detection of SARS-CoV-2. In addition, we discuss principles, advantages, and disadvantages of these detection methods, and highlight the potential methods for the development of additional techniques and products for early and fast detection of SARS-CoV-2.

## 1. Introduction

At present, the rapid worldwide outbreak of SARS-CoV-2 infection and associated coronavirus disease 2019 (COVID-19) is impacting human and economic health. Because community spread occurs easily, the number of infected individuals has been constantly mounting [1]. Responding to this rapid transmission requires rapid identification of the infected individuals, regardless of symptoms. One challenge is that asymptomatic individuals can spread the virus. While symptomatic individuals are the most common source of transmission, undiagnosed asymptomatic cases increase the risk of transmission and therefore, increase COVID-19 infection [2,3]. Prevention, detection, control, and treatment of COVID-19 are linked, and each aspect of COVID-19 management influences the others. 

Accurate and early detection of SARS-CoV-2 is vital to decrease the risk of transmission by rapidly enabling isolation and contact tracing. Consequently, the most significant public health impact comes from the rapid detection of infected cases. 

Clinical detection of COVID-19 is principally based on clinical symptoms and history of contact with other possibly infected individuals. Since the clinical manifestations and signs of infected patients (pneumonia, dyspnea, fever, cough, respiratory symptoms) are not definitive [4], supporting diagnostic and serological tests are essential for the diagnosis of COVID-19. The value of diagnostic methods depends on the type of test, the time to get the results, testing accuracy, and the required resources for testing. In other words, the quick identification of suspected individuals is the best strategy to enable appropriate response and limit transmission.

Different diagnostic tests have been developed for SARS-CoV-2 based on serological, molecular, and nanotechnology techniques. Detection of viral nucleic acid is frequently performed by high-throughput sequencing, reverse-transcription-polymerase chain reaction (RT-PCR), RT-loop-mediated isothermal amplification (RT-LAMP), and quantitative real-time PCR (qPCR) [5,6,7], where qPCR is recommended as the most effective and direct method by the WHO (World Health Organization). Immune methods such as detection of SARS-CoV-2-specific IgM/IgG can identify previous or current infection [4,8]. Here, we discuss current knowledge on the diagnosis of COVID-19 that can be useful in suggesting novel insights for battling the SARS-CoV-2 infection. We have used PubMed, EMBASE, Scopus, and Google scholar databases for studies. The search terms used were “coronavirus”, “COVID-19”, “SARS-CoV-2”,, “diagnosis”, “detection”, “nanotechnology”, “serological testing”, “sensor”, and “Point-of-Care Detection.” The reference lists of the eligible articles were also reviewed to search for relevant articles. We included preprint, peer-reviewed, and retrieved full-text articles and examined the citation chain for each article to be included.

## 2. Serological Approaches in the Detection of SARS-CoV2

A diagnostic method to identify an antibody-mediated immune response against infectious agents is termed serological testing [9]. However, this procedure does not identify the virus but determines whether an individual is or has been infected, by identifying an antibody immune response against past or current infections. Since it does not detect the early phase of infection, the European Center for Disease Control and Prevention (ECDC) has endorsed COVID-19 serological testing for epidemiological and surveillance means only [10]. Identification of preceding infections even without testing in the active phase of the disease is possible with these techniques and is the main advantage of serological approaches. COVID-19 has been detected with several serological tests, some of which were marketed as point-of-care and rapid methods so far. However, test accuracy results remain challenging [11]. The serological test of COVID-19 provides information on the type and concentration levels of various immunoglobulins (IgA, IgM, and IgG) produced due to infection by SARS-CoV-2. Still, with novel COVID-19s late emergence, recently published data, studied by Guo et al., informs on antibody’s median appearance time in plasma after the onset of symptoms. Accordingly, IgM and IgA require 3–6 days and IgG requires 10–18 days to develop with positivity rates among known COVID-19 patients of 85.4%, 92.7%, and 77.9% for IgM, IgA, and IgG, respectively [12]. Slightly different results come from another recent study on the kinetics of anti-COVID-19 antibodies conducted by Padoan et al., with the appearance of IgM and IgG at 6–7 days after symptoms onset [13]. Surprisingly, with anti-SARS-CoV-2 IgG development on 100% of patients 12 days after symptoms onset, less than 90% of the same group developed IgM. 

As stated by the authors, within two weeks after the onset of symptoms, anti-SARS-CoV-2 antibody positivity was nearly 100% for both IgA and IgM, whereas IgG has been positive for only 60% of the same patients [14]. Another supporting study conducted by Zhang et al. reported anti-SARS-CoV-2 IgM positivity of 50% and IgG 95% [15]. Also, according to Du et al.’s study, anti-SARS-CoV-2 IgM and IgG antibodies’ rate in convalescents is 78% and 100%, respectively [16]. Pen et al. could find anti-SARS-CoV-2 antibodies positivity 15 days from symptom onset, approximately 74% for IgM and 97% for IgG [17]. SARS-CoV-2’s potential to provoke IgA secretion even in mild or asymptomatic forms of COVID-19 has been a recent noteworthy point in the context of improving the diagnostics, following their determination in blood and saliva [18]. 

Anti-SARS-CoV-2 antibodies’ effectiveness on neutralizing and pathogenesis of the virus, as measured by their blood presence, has been a challenging issue. Recent publications show auspicious data on supporting the antibodies to target nucleocapsid and spike proteins, and consequently neutralizing virus effects [19]. Additionally, according to Okba et al.’s study, COVID-19 patients’ serum is capable of neutralizing the SARS-CoV-2 infection [20]. The significance of antibodies’ concentration persistence necessitates reviewing past experiences. Conclusions from former and related coronavirus disease SARS informs the persistence of anti-SARS-CoV-1 neutralizing antibodies in the blood. Provided information claims that antibodies were highly stable for 16 months after infection following an eventual decline to 50–75% after 4 years and ~10% after 6 years [21]. A final concern with COVID-19 is the anti-SARS-CoV-2 immunoassays’ potential to cross-react with previous coronaviruses such as SARS-CoV-1, MERS-CoV, HCoV-HKU1, HCoV-OC43, HCoV-NL63, and HCoV-229E.

A mobile non-automated method for evaluating membrane-based immunoassays and extracting qualitative data rapidly, approximately 5–20 min, is a rapid serological test. The advantages include low sample volume (a blood drop is sufficient), brief operator training, cost-effective testing, and simple instructions. Besides, usage is common in bedside or near-to-patient situations [22]. Rapid serological testing has two main approaches. First, identifying SARS-CoV-2 antigens, and second, determining anti-SARS-CoV-2 antibodies. The ECDC provides a steadily updated and appreciating description of the procedure [23]. Spain and other European countries’ criticism of these tests’ inaccuracy and doubtful obtained diagnosis and surveillance conveyed skeptical aspects toward the performance of test kits [24]. Furthermore, evidence concluded from Cassaniti et al.’s study states a rapid test sensitivity below 20%, consequently resulting in a large-scale COVID-19 under-diagnosis [25], making this technology unusable if that analysis is correct. Thus, every device must undergo a validation process before its clinical application. The suggestion in parallel with ECDC is providing scientific publications to elucidate the performance and limitation of each rapid diagnostic test before their presentation to diagnostic and clinical management fields, public health, and epidemiologic surveillance [23,26]. It should be understood that rapid diagnostic tests primarily contribute to supporting decentralized testing capacity, rather than a replacement for central laboratory diagnostics [27]. Assay by standard clinical labs uses venipuncture blood collection instead of capillary blood collection and it has a crucial dependency on laboratory analyzers’ participation. In contrast to all these drawbacks, it has advantages such as accuracy and reliability, generating quantitative data essential for longitudinal titer monitoring, expert laboratory personnel performance leading to mitigating error risks and subjective interpretations, permanent test results’ storage in the LIS (laboratory information system), and following strict internal quality control and presumably, external quality assessment schemes (EQAs) in the future. The modern generation of laboratory analyzers has outstanding sample flow rates of up to several hundred tests per hour, with respect, centralized laboratory diagnostic methods are convenient and efficient for epidemiologic surveillance means. Intriguingly, the University Hospitals of Padova and Verona (Italy) have been worldwide pioneers in appreciating and advancing an approved project by the scientific committee of the Veneto Region. They are engaged in a broad, validated, fully automated immunoassays’ epidemiological screening of healthcare professionals in the Veneto region (i.e., between 50,000 and 70,000 people). The project’s phase 2 indicates the possibility of widening the analysis to nearly 5 million residents of the whole Veneto region [28].

## 3. Molecular Approaches for Detection of SARS-CoV-2 Infection

Many companies and research groups have developed diagnostic tools for this single-stranded, positive-sense RNA virus. The complete genetic sequence of the virus was uploaded to the Global Initiative on Sharing All Influenza Data (GISAID) platform, which has provided the information needed for molecular detection of the genome. 

### 3.1. Reverse Transcription-Polymerase Chain Reaction (RT-PCR)

Amplification of very small amounts of viral genetic material in a mixture of other nucleic acid sequences is effectively done by RT-PCR and is currently the gold standard technique of SARS-COV-2 detection in upper respiratory tract samples. Additionally, a few studies have utilized serum, ocular, and stool specimens for the RT-PCR-based detection method [29,30,31]. A recent method has used self-collected salivary samples as a non-invasive and safe technique for healthcare providers before doing RT-PCR [32,33]. In this method, the reverse transcriptase first converts the RNA viral genome into DNA with the use of a small DNA sequence primer and the final generation of the complementary DNA (cDNA). A fluorescent dye or a fluorescent-labeled sequence-specific DNA probe monitors the amplification of DNA in real-time. A fluorescent or electrical signal reveals the viral cDNA after consecutive amplification cycles [34]. 

Traditional RT-PCR techniques contained one-step or two-step processes. While one-step methods involve a single primer-contained tube, the two-step procedure uses more than one tube to run the reactions but provides a more sensitive and flexible pathway. Also, it can stock cDNA for quantification of various targets with less starting materials [35]. However, the common method in the detection of SARS-CoV-2 is the one-step approach since it is faster, requires less sample handling, decreases bench time, and reduces pipetting errors. Several SARS-CoV-2 genomic regions such as ORF1b or ORF8 regions, and the nucleocapsid (N), RNA-dependent RNA polymerase (RdRP), spike (S) protein, or envelope (E) genes, have been used in molecular diagnosis of the virus via RT-PCR technology [36,37]. COVID-19 RT-PCR (LabCorp), 2019-Novel Coronavirus Real-Time RT-PCR Diagnostic Panel, TaqPath COVID-19 Combo kit (ThermoFisher, Applied Biosystems), Allplex 2019-nCoV Assay (Seegene), and cobas SARS-CoV-2 (Roche) have been the utilized commercial assays so far [6,35,36]. Also, more automated techniques and detection tools have improved the utility of RT-PCR tests. For instance, GenMark *Diagnostics* Inc., which uses “The True Sample-to-Answer Solution” ePlex apparatus developed to detect SARS-CoV-2 in nasopharyngeal samples [38]. The viral RNA is extracted by a magnetic solid-phase procedure and all other reagents required for cDNA amplification are found in each test cartridge. Also, a combined GenMark’s eSensor technology and electrowetting method are used to detect the virus. Although this method has extensively been applied in the detection of COVID-19, some problems such as costly required equipment, incorrect sampling, expert personnel, and limitation in sample transfer lead to delayed results. Thus, the improvement of the RT-PCR method by addressing these limitations is an important issue to be solved [39]. 

### 3.2. Isothermal Nucleic Acid Amplification

The requirement for sophisticated thermal cycling equipment is a limitation for RT-PCR techniques [40]. Using isothermal nucleic acid amplification eliminates this requirement and allows amplification at a constant temperature. Different approaches have been developed based on this strategy. Reverse Transcription Loop-Mediated Isothermal Amplification (RT-LAMP) has been introduced as an easy and cost-effective method to detect SARS-CoV-2 which uses a series of 4 target-specific primers to augment test sensitivity in a combined LAMP and reverse transcription-based methodology. The measurement of turbidity induced by magnesium pyrophosphate as a byproduct of the amplification process is performed by photometry. Then, both photometric and/or fluorescent assays can be utilized in real-time. The need for only heating and visual inspector steps turns RT-LAMP into a rapid and sensitive tool in virus detection [41]. Currently, Abbott *Diagnostics* uses RT-LAMP in SARS-CoV-2 detection as a point-of-care setting in nasal swabs. However, it is restricted to one sample/run [37,42]. Also, the colorimetric LAMP can detect viral RNA in cell lysate samples at levels of about 481 RNA copies lacking interferences, which is a promising rapid diagnostic approach for SARS-CoV-2 RNA [6]. 

The other isothermal amplification strategy is transcription-mediated amplification (TMA), which can amplify specific regions of both RNA and DNA [37]. TMA uses T7 RNA polymerase combined with a retroviral reverse transcriptase enzyme. Accordingly, Hologic’s Panther Fusion platform can perform both RT-PCR and TMA [43]. High testing output and simultaneous screening of common respiratory viruses with similar symptoms of COVID-19 are the main advantages of the Panther fusion platform. Hybridization of the viral RNA target with a specific capture probe and an extra T7 promotor primer, which are captured via a magnetic field, commences the reaction. Afterward, the reverse transcription of T7 promotor primer-bound captured RNA to a complementary cDNA is performed. The activity of RNase reverse transcriptase consequently results in degradation of the target RNA strand while producing a T7 primer including single-stranded (ss) cDNA from an RNA–DNA hybrid. Also, T7 RNA polymerase is used to produce RNA amplicons with the application of additional primers. These amplicons reenter the TMA process, which ultimately leads to the generation of billions of RNA amplicons in a short time. The ss nucleic acid torches which are bound to a fluorophore and a quencher are used in the detection process. The hybridization of torches to RNA amplicons in real-time results in the emission of a signal from the fluorophore. 

CRISPR has been developed for the detection of SARS-Cov-2. The use of Cas nucleases (Cas12 and Cas13) enables CRISPR-based detection techniques [44,45,46]. Cas13 has been harnessed in RNA/DNA detection in an approach called SHERLOCK as a non-specific RNase [44]. Amplification of the target RNA by a combination of T7 and RT-RPA transcription processes is the first step in the SHERLOCK method. This, in turn, activates Cas13, which subsequently cleaves a reporter RNA that releases the fluorescent dye from a quencher. The CRISPR-nVoV has used the SHERLOCK method in the detection of SARS-CoV-2 RNA with great sensitivity in 52 patient specimens [47]. Cas12 as an RNA-directed DNase cleaves ssDNA from a target sequence in a method termed DETECTOR [45]. Several groups have used this method in the detection of SARS-CoV-2 recently. Isothermal amplification of viral RNA after its conversion to DNA is the initial step. Then, the Cas12 is activated by specific target sequences in amplified DNA and subsequently cleaves an ssDNA reporter to unquench a fluorophore. The CRISPR-based method can yield rapid read-outs and sensitive results when used in combination with fast isothermal amplification processes. Also, they could be coupled to lateral flow readouts which are suitable candidates for simple point-of-care testing approaches. Low turnaround timeframe, high sensitivity, and less bias generation are considered as the advantages of this method but expensive equipment, expert personnel, and sampling limitations should also be taken into account [39].

### 3.3. Nucleic Acid Hybridization Using Microarray

Efficient and sensitive detection of SARS-CoV nucleic acids have also been performed with microarray assays. Generation of cDNA from viral RNA, which are then labeled with specific probes, commence the microarray assays. Solid-phased oligonucleotides fixed microarray trays are used to load labeled cDNAs. The presence of viral-specific nucleic acid will be shown if the hybridization process occurs [48]. The mutations and single nucleotide polymorphisms related to the SAR-CoV gene have been successfully determined with microarray assays [49]. This would help the rapid detection of different COVID-19 strains and mutational variations. Portable microarray chips have provided efficient identification of the MERS coronavirus in addition to influenza and respiratory syncytial viruses [50]. Microarray techniques that use a scanner to show the hybridization between the probe and target are quite rapid, sensitive, specific, and accurate means of detection. They can also analyze several microbial genes concurrently. Although it can detect multiple samples, diagnosis of a few viral genes in limited samples is not possible with this method [51].

### 3.4. Amplicon-Based Metagenomic Sequencing

Combinational use of amplicon and metagenomic sequencing has been applied in the detection of SARS-CoV-2, termed Amplicon-Based Metagenomic Sequencing. Metagenomic sequencing was initially utilized to determine the related microbiome of infected individuals. The potential contact tracing, viral evolution investigations, and molecular epidemiologic studies are assessed by amplicon-based sequencing. Additional analysis on sequence divergences is provided by metagenomics tactics such as sequence-independent single primer amplification (SISPA). The examination of the mutation rate of SARS-CoV-2 and other related recombinants could be determined with this dual technique. Moore et al. used MinION sequencing for rapid SARS-CoV-2 sequencing and other upper respiratory system swabs [52]. Illumina has provided a next-generation shotgun metagenomics sequencing platform that not only diagnoses different coronavirus strains but also, can check other organisms present in a complex sample [37]. The presence of comprehensive reference databases, available patterns for bioinformatics tests, and detecting rare taxa Taxonomy of the gene level are the advantages of this method. Biases in vial population quantification are the main disadvantage of this technique [53]. 

## 4. Point-of-Care Detection of COVID-19

**Figure 1:**
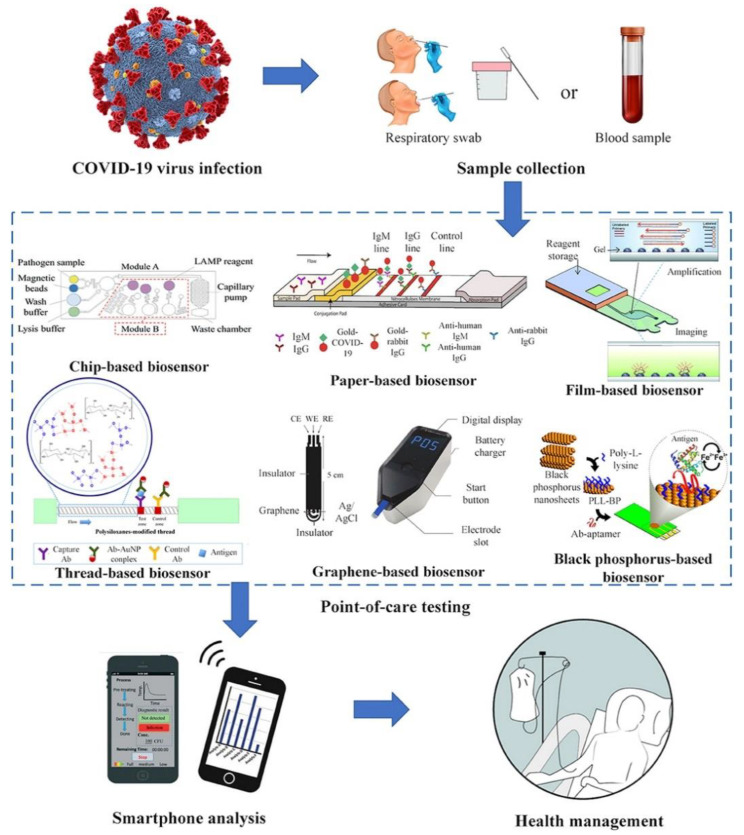
Point-of-care biosensors for coronavirus disease 2019 (COVID-19) (Adapted from Reference [54] with the permission under the terms of Creative Commons Attribution License).

The point-of-care biosensors potentially utilized for COVID-19 include sample-to-answer chip-based biosensors, paper-based biosensors, or other material-based biosensors, as shown in Figure 1. 

Diagnosis of patients in the absence of centralized lab facilities is called point-of-care testing. Lateral flow antigen identification for SARS-CoV-2 is developed as a point-of-care testing methodology toward diagnosing COVID-19 [55]. Regarding chip-based biosensors, two constituent lines of commercial lateral flow assays are composed of gold nanoparticle antibody conjugates and capture antibodies, respectively. Deposited blood or urine samples’ proteins on the membrane transit by capillary action. On the primary line, the antigens attach to the gold nanoparticle–antibody conjugates, and as complexes reach the second line, they become immobilized by capture antibodies. Eventually, red and blue lines appear. Red lines are presented as gold nanoparticles exclusively, and blue lines as a clustered gold solution on account of plasmon band coupling. The lateral flow test’s accuracy, specificity, and sensitivity have been demonstrated 69%, 57%, and 100% for IgM and 86%, 181%, and 100% for IgG, respectively. The simultaneous detection of IgM and IgG produces a clinical sensitivity of 82% [55]. Lateral flow assay tests can expand into nucleic acid testing. Combination of the RT-LAMP test with lateral flow assay to determine MERS-CoV was formerly experienced [56]. However, these tests are one-time use and have deficient analytical sensitivity in contrast to RT-PCR. To compensate, researchers have developed signal amplifying methods such as thermal imaging and multiple gold nanoparticle assembling [57]. Also, a microfluidic device as an alternative facility to utilize the point-of-care testing is composed of a palm-sized chip with micrometer-sized channels and reaction chambers. Mixing and separation of liquid specimens in chips occur due to electrokinetic, capillary, vacuum, and other forces. Some advantages of utilizing microfluidics are miniaturization, small sample volume, rapid detection times, and portability [58]. Laksanasopin et al.’s endeavor has brought up a microfluidics-based smartphone add-on that detects antibodies against three sexually transmitted infections by sequentially moving reagents pre-deposited on a cassette. A performed test of the platform on 96 patients in Rwanda illustrated sensitivity and specificity for HIV as 100% and 87%, respectively [59]. The stated technologies have the potential to detect SARS-CoV-2 RNA or proteins. 

Due to their early-stage developing status, such approaches are not trusted for the early diagnosis of COVID-19. Phase 1 presents technologies in the proof-of-concept stage, in which synthetic targets are used by researchers for concept validation. Phase 2 refers to technologies undergone analyzing limited patient samples (i.e., <100 samples). Phase 3 introduces technologies advanced to large patient cohort clinical trials. Phase 4 offers technologies that are commercialized and utilized in patients. Such edge tools probably play a role in the diagnosis of upcoming diseases.

In addition to chip-based and paper-based biosensors, other material-based biosensors such as textile-based, film-based, or carbon-based biosensors have also been presented for possible implementation for COVID-19 [60,61]. They are developed to enhance the functionality and detection sensitivity of the current biosensors, with a better perspective in clinical settings. 

Biosensors are reproducible, easy, rapid, and sensitive means of detection which need a small sample size and could be miniaturized [62]. The awareness of patients and other consumers before the application of this test is considered as a drawback since the point-of-care system might not meet the required standards of accreditation as laboratories do [63].

## 5. The Role of Smartphone in the Detection and Surveillance of COVID-19

Control of an epidemic demands vast surveillance, exchanging data, and patient monitoring [64,65]. Healthcare’s proper function in all contexts from a local hospital to WHO, claim assistance tools in promptness, and communication simplicity to restrain disease spread. Due to smartphone’s connectivity and computational power potential, they have been predisposed as hardware to simplify electronic reporting, epidemiological database, and point-of-care testing (Figure 1) [66,67]. Smartphones as a readily accessible tool all around the world (including sub-Saharan Africa) accommodates them to organize responses during huge epidemics like COVID-19 [66]. Incompetence in communication and reporting has facilitated the global spread of COVID-19 [68,69]. Iran, as a vivid instance, had 43 confirmed cases by 23 February 2020, a fatality rate of 19%, and 3 exported cases. According to this report and transmission modeling, the approximate estimation of infected individuals was thousands [70]. Smartphones’ pairing with prior diagnostic tests affords real-time geospatial data, which prompts national and global health agencies to conduct synchronized regulatory stratagems. Former experiences of using smartphones as a geospatial infectious tracking tool are also accessible in research groups’ endeavors to tackle HIV, Ebola, and tuberculosis [71,72,73]. In the Ebola outbreak, smartphones were utilized as a contact tracing tool, facilitating tracking and identifying people in contact with the patient [71]. Smartphones’ contact tracing produces broader data with sharing options. In the absence of regional healthcare agencies’ communication, transmission rates differ across a country [74]. Indeed, through the 2003 SARS outbreak in Canada, Toronto, Ontario had 247 cases, with 3 imported ones, and Vancouver, British Columbia had only 5 cases, with 4 imported ones [74,75]. Although Ontario suffered from a lack of provincial public health agency, British Columbia noticed an upcoming threat of importing emerging infectious diseases. Therefore, the public health agency of British Columbia developed a digital network communication over the province [75]. Smartphone connectivity widens these communication networks. Smartphones provide the possibility to upload and share epidemiological data with public health databases and also manage outbreak responses. Suspected COVID-19 cases may confront communication difficulties and anyone indicating mild respiratory symptoms faces barriers in traveling to overcrowded hospitals, due to the increased risk of contact with possible COVID-19 patients. Smartphones ease patient and clinician contact without disease spread risk. Also, throughout the 2009 influenza pandemic, Switzerland, despite not possessing a reporting system yet, deployed medical teleconsultations to control suspected cases [76,77]. Teleconsultations generated more influenza reports in contrast to in-person consultation due to obstacles in reaching out to people. COVID-19-infected patients, upon testing positive and having mild symptoms, are sent home for self-quarantine [77]. Self-quarantine innately hinders the patient’s contact with clinicians, leading to monitoring challenges and detrimental mental health effects. Smartphone apps assist patients to stay in touch with mental health counselors to cater to their needs during isolation, disease outbreak, and self-quarantine [78,79]. Besides, patients can self-report symptoms and behaviors contributing to remote monitoring by clinicians [80]. Smartphone-linked reports inform epidemiologists on potential transmission mechanisms. For instance, at the time of the 2013 MERS outbreak, a smartphone application facilitated monitoring travelers during their Hajj pilgrimage. Application users were aware of hand hygiene protocols and reported animal contact, and the onset of symptoms, both during the pilgrimage and after it [81]. Such similar applications are available to provide constant information for public health agencies and consequently improve their response toward disease outbreaks. Recently, cooperation between smartphone and diagnostic technologies has had considerable advances. Furthermore, smartphone components such as a camera, flashlight, and audio jack have been a substitute for conventional laboratory equipment in reading out diagnostic assays [82]. Smartphones automate readout and database aid, and diagnostic procedure. As a practical purpose, a smartphone-based microscope went through a field test in Cameroon and illustrated faster turnaround times with respect to the standard techniques [83]. Kanazawa et al. endorsed utilizing smartphones by using forward-looking infrared radar (FLIR) for detecting thermal variations due to inflammation. Additionally, this method may also aid in detecting fever as a general sign of many coronavirus infections, including COVID-19 [84]. Mudanyali et al. established a smartphone-based microscope that conveys diagnostic outcomes to a database for analyzing and spatiotemporal mapping [85]. At the community level, where reporting encounters challenges, these devices provide addressing the requirement for point-of-care testing.

## 6. Wastewater-Based Epidemiology

The majority of the population infected with the SARS-CoV-2 virus remains asymptomatic or illustrates mild symptoms of infection. These symptom-free carriers increase the risk of disease transmission, especially in the lack of appropriate quarantine policies and preventive actions during the pandemic of COVID-19. The preventive actions at the early stage of a pandemic should include the rapid recognition of obscure sources and widespread screening for fast detection of asymptomatic cases [86]. Due to the high transmission rate of this virus, sieving of the infected cases by medical staff needs huge amounts of preventive equipment. Moreover, these screening tests are not accessible in most infected areas due to economic problems. Therefore, the substitutional wastewater-based epidemiology (WBE), which was successfully applied for the detection of illicit drugs or various pathogens, could be a promising approach for anticipating spread of the virus [87]. This virus is detected in feces and urine samples of infected patients, therefore the sewer system, as an indicator and holder of various biomarkers of diseases in the community, could be used during the pandemic of COVID-19 [88]. Due to different reports about the isolation of the active form of this virus in urine and feces and based on the remarkable survival duration of this virus in a suitable situation, detecting the most infected regions could be possible by analyzing the community wastewater system [86,88]. Therefore, it could be concluded that analyzing community wastewater could determine the local regions with a high incidence of SARS-CoV-2. This determination in the early stages is more effective and leads to rapid and comprehensive actions to reduce the diffusion of the virus in the target region. Use of the WBE method needs a rapid, effective, and cost-benefit strategy for virus detection. The polymerase chain reaction (PCR) is the common method for the detection of virus DNA. However, this method has some limitations such as complex and time-consuming sample preparation and analyzing method, which needs expensive instruments and well-trained technicians. Therefore, other suitable and transportable methods should be considered for evaluating wastewater on-site and detecting COVID-19 by WBE [86]. Paper analytical gadgets are a promising alternative in the rapid detection of pathogens to overcome the limitations of the PCR method [89]. For example, the application of this paper for the detection of malaria from whole blood leads to a faster and more effective diagnosis of this pathogen compared with the conventional PCR method. In this gadget, all of the DNA detection procedures are gathered in one paper. This contraction leads to an inexpensive, rapid, efficient, and easy method for virus detection [90]. These papers are user-friendly and could easily be used by people due to clear contrast with a colored substrate and facility of transportation to the site of examination [86]. The application of these papers in pathogen detection in wastewater has been approved previously [87]. 

SARS-CoV-2 is a non-enveloped enteric virus, which is expelled in feces less than noroviruses and demobilized faster than other non-enveloped ones in wastewater. Moreover, the specific genomic structure of COVID-19 (large ssRNA) increased the degradability of this virus under UV radiation. Therefore, the current wastewater treatment system in developed countries is completely effective in the elimination of this virus. However, the sewage infrastructure or using the wastewater for irrigation besides non-efficient plumbing system increased the risk of virus diffusion in some regions [91,92,93]. Based on investigations on various species of RNA viruses, most of them are enveloped and most of the future studies should focus on their nature, inactivation, and diffusion due to the wide diversity range in this family [91,94,95,96]. Therefore, further investigations in future studies should be considered and these trials should use a standard virus species (e.g., bacteriophage MS2) for standardization of the analyses [90,96,97]. Therefore, better recognition of the enveloped-virus fate and expelling in feces and urine, as well as other pathogenic microorganisms, leads to employing wastewater-based detections to control the diffusion of the virus in society in premature point of clinical symptoms’ onset [90,98,99].

Several benefits could be obtained from wastewater surveillance of COVID-19. It can prohibit biases of other epidemiological markers in a cost-effective way via collecting the information of individuals who lack access to healthcare. It can provide near-real-time data of the infection prevalence before disease diagnosis [97].

## 7. Nanotechnology-Based Approaches in the Detection of COVID-19

Nanoscience and nanotechnology are studies that deal with very small particles and are used in many other disciplines, such as chemistry, biology, physics, materials science, and engineering [3,98]. Many scientists believe that the best way for controlling the spread of COVID-19 is to diagnose the spread of the virus quickly, cheaply, reliably, and agilely using novel nano-systems until the vaccine is detected [99]. 

**Figure 2:**
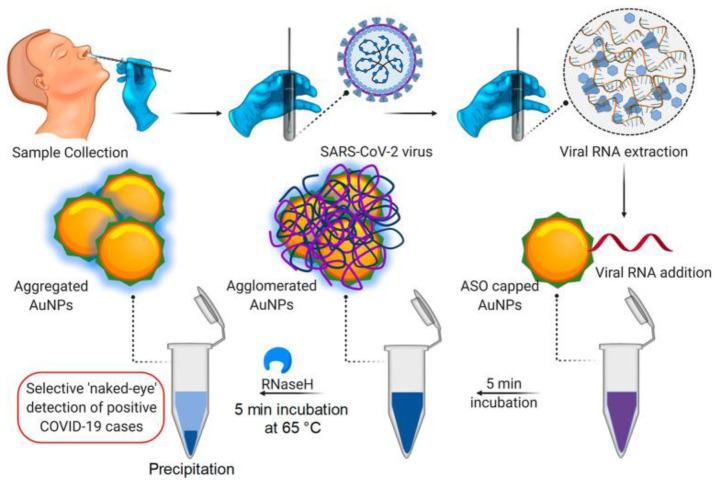
Selective Naked-Eye Detection of SARS-CoV-2 RNA Mediated by the Suitably Designed AuNPs (Adopted from Reference [100] with permission). https://pubs.acs.org/doi/abs/10.1021/acsnano.0c03822, further permissions related to the material excerpted should be directed to the ACS.

**Figure 3:**
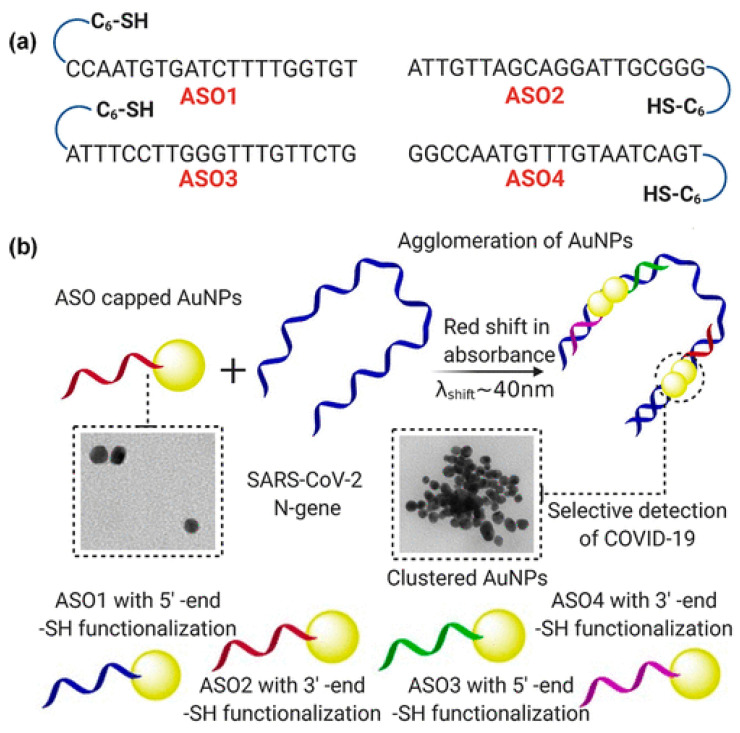
In (**a**) antisense oligonucleotides (ASOs) are shown with their different sequences and functions. (**b**) Schematically shows the proposed idea of accumulation of gold nanoparticles when capped with the ASOs (Adopted from Reference [100] with permission). https://pubs.acs.org/doi/abs/10.1021/acsnano.0c03822. Further permissions related to the material excerpted should be directed to the ACS.

Scientists have shown that the combination of the use of advanced nanomaterials and protein detection for each disease can have a positive effect on the rapid diagnosis of various diseases. Moitra and coworkers [100] have developed a test to diagnose COVID-19 that can detect the virus in 10 min (Figure 2 and Figure 3). The diagnostic method is very simple and due to the presence of plasmonic gold nanoparticles, the test is positive by changing the color. This test does not require complex laboratory methods such as DNA analysis. This method can detect the RNA of the virus on the first day of infection. Upon receipt of saliva from the patient’s mouth or nasal mucosa, RNA is extracted from the sample within approximately 10 min. The test is performed using nanoscale gold molecules to detect specific proteins. When the biosensor is connected to the virus’s gene sequence, the gold nanoparticles change the color of the liquid reagent from purple to blue. The accuracy of the COVID-19 test depends on the ability to discover the virus. That is, if the virus is present, the negative result will not be wrong, and if the virus is not present, the positive result will not be wrong. Many test methods available in the market are not able to diagnose the disease for several days after infection. That is why many of the negative responses that come with these tests are wrong. The authors believe that the cost of producing and using this test is much cheaper than the laboratory tests because it does not need a laboratory apparatus or skilled people to perform and examine it. This method meets the requirements of the FDA. This method can be used in any area, including daycare centers, nursing homes, college campuses, or work places [100].

In addition to diagnosing COVID-19, we need to be able to identify patients at high risk of death (people with cardiovascular disease, severe respiratory illness, or severe lung damage). This frees up the capacity of medical care centers so that they can react quickly, saving many lives [101]. Based on a report by Mahmoudi et al., to inhibit severe lack of healthcare systems, diminish death rates, and advance control of future epidemics and pandemics of COVID-19, two main areas for the detection based on nanotechnology can be used: biomolecular corona and magnetic levitation. The idea of both technologies is adopted from the varying levels of infection and phases of disease which change the composition of biological fluids in the body of the host and can act as a fingerprint [101]. 

As we know, nanoparticles can bond to a variety of biomolecules, including proteins, as soon as they enter the biological environment, i.e., human blood. To perform this test, a patient’s biological fluid is introduced to a small collection of nanoparticles. The surface of these nanoparticles is covered with biomolecules—the so-called biomolecular corona—and will give this nanoscale a unique and completely different biological identity. Then, by investigating the composition of the crowns at the surface of the nanoparticles combined with statistical methods, the results may present a ‘fingerprint’ pattern for patients who might be at risk of death after being infected by COVID-19 [101,102,103]. This method can also be used to accurately diagnose the deadly and non-lethal types of Coronavirus [104]. Technology of protein corona sensor array can be useful to define the plasma protein or biomolecule patterns that imply deadly COVID-19 infection at the very beginning. Although much of the biomolecular corona is covered in protein, there are other biomolecules (metabolomes, lipids, nucleic acids) that are effective in diagnosing corona [101].

In the case of rapid diagnosis or home testing, the bioavailability of rapidly accessible biological fluids, such as urine, tears, or saliva, can be considered in the protein corona sensor array method. These fluids contain protein markers associated with the disease. Compared to human plasma, which requires blood sampling, the purpose of using the aforementioned biological fluids is different in that a device can be developed where non-specialists can perform diagnostic procedures. However, the disadvantage of this method is that the biomolecules in biological fluids are much lower than in plasma. Therefore, the accuracy of the diagnosis is reduced [105].

Their groups also suggested another method dependent on magnetic levitation (MagLev) of nanoparticles. In this strategy, the patient plasma samples suspend in a solution of magnetic nanoparticles. Then, the distinct bands of proteins form over time, separated by density. Much like the protein crown, these distinctively shaped bands of proteins make distinct and reliable patterns valuable for fingerprinting disease and phases of infection. The MagLev method for measuring protein concentrations provides useful information to better understand the biochemical properties of proteins. Recent research has shown that levitation patterns belonging to human blood plasma proteins provide valuable information about the health spectrum of donors. Because different diseases cause different changes in the plasma proteome, levitation progress and patterns of plasma proteins will provide valuable information about a person’s health status [101].

The main weak point of both biomolecular corona and the MagLev method is that there are no biomarkers or nucleic acids for diagnosis. Therefore, in the first stage, we must collect plasma and non-plasma biological fluids from a significant number of people with COVID-19 in the normal and severe stages of the disease. Then, the data obtained from the testing of these liquids should be analyzed by omics and machine learning methods, and its biomolecular patterns, which are closely related to the high risk of death in this disease, should be determined. On the other hand, the main advantage of these methods compared to conventional diagnostic methods is the ability to detect extensive different types of biomolecular patterns. This feature will be effective in quickly and accurately detecting deadly COVID-19 infections. The main reason is that many biomolecules are associated with personalized plasma variation or co-morbidity [104,105]. Nanomaterials can provide new opportunities such as more effective, convenient, and safer applications. However, challenges such as costs, toxicities to the environment and humans, and regulatory issues should be solved before introduction to the market [106].

Table 1 presents methodologies, their principle, needed samples, cost, advantages, and massive used methods in the detection of SARS-COV-2.

## 8. Future Perspectives

The aid of technology has offered great innovations in disease diagnostics. However, to obtain better results, the integration of recent development is crucial. The easy, effective way of liberation and enrichment of COVID-19 virus RNA requires more research. Also, the applications of combined technologies, such as highly sensitive detection tolls like biosensors with effectual isothermal RNA amplification, is required to achieve real-time and sensitive detection.

While the timeframe for virus-related antibody formation is 1–2 weeks, virus particle detection is preferred in the early stages of the disease via point-of-care screening methods. However, body fluids contain low levels of virus particles. Thus, the use of novel detection techniques as described here is useful. Development of a self-consistent point-of-care apparatus that accurately detects the virus and can test the infection progression still needs more improvements. This method could provide more efficient systems in the detection of the diseases in patients with a higher risk of death. 

## 9. Conclusions

This manuscript aimed at reviewing the detection methods of COVID-19 as a global health concern. All the above-described techniques can be effectively deployed for the detection of COVID-19 in different settings. For example, real-time RT-PCR and serological methods are still the most extensively used detection techniques in large hospitals, while the biosensors, point-of-care testing, nanotechnology-based approaches, smartphone surveillance of infectious diseases, amplicon-based metagenomic sequencing, and smartphones are still expected to be further developed as large-scale screening techniques that can even in some cases, such as biosensors, be used in the home settings. 

## Figures and Tables

**Table 1:** A summary table with all the methodologies, their principle, needed samples, cost, advantages, and massive used methods.

Methodologies	Test	Principle	Sample	Advantage	Massive Used	Cost
Serological approaches	Enzyme-linked immunosorbent assay (ELISA)	Binding of antibody against COVID-19 with coated antigen in ELISA plates to forming complex and detect with labeled secondary antibody which produced color or fluorescence.	Blood serum or plasma	- Rapid	✓	Not very expensive
	Chemiluminescence immunoassay (CLIA)	By chemical probes which could produce light emission via chemical reaction to label the antibody against COVID-19.	Blood serum or plasma	- Sensitive - Rapid		Expensive
	COVID antigen assay	Detection of COVID-19 antigen with its specific antibody based on ELISA or CLIA	Blood serum or plasma	- Rapid		Variable costs
Molecular approaches	RT-PCR	Conversion of RNA of COVID-19 to cDNA via transcriptase enzyme followed by real-time PCR for amplification of cDNA	Upper respiratory specimens	- Gold standard test - Sensitive	✓	Expensive
	RT-LAMP	Conversion of RNA of COVID-19 to cDNA via transcriptase enzyme and is performed at a temperature between 60 and 65 °C.	Upper respiratory specimens	- Does not require thermal cycler - Time efficient - Does not need access to high-tech laboratory		Very cost effective
	Nucleic Acid Hybridization Using Microarray	Conversion of RNA of COVID-19 to cDNA via transcriptase enzyme followed by adding it in wells containing fixed COVID-19-specific oligonucleotides then washing the hybridized virus cDNA for remains and emitting signal for positive samples.	Upper respiratory specimens	- Sensitive		Expensive
	Amplicon-Based Metagenomic Sequencing	Hypervariable regions of conserved genes or intergenic regions are amplified by PCR, evaluated by the next-generation sequencing (NGS), and the resulting sequences are compared against databases.	Upper respiratory specimens	- Diagnoses of the different coronavirus strains		Expensive
Point-of-Care detection of COVID-19	Lateral flow assays	The antigens bind to the gold nanoparticle–antibody conjugates. The red and blue lines appear. The red lines are presented as gold nanoparticles exclusively, and blue lines as a clustered gold solution on account of plasmon band coupling.	blood or urine	- High specificity - Rapid - Simple and easy to use - No need for a laboratory		Cheap
	Biosensors	Based on type of sensor, its principle is different.	Upper respiratory specimens or blood or urine	- Sensitive - Rapid - Easy to use		Expensive
Nanotechnology-based approaches	The application of nanoparticles in several methods which are mentioned above	Based on type of method, its principle is different.	Upper respiratory specimens or blood or urine	- Sensitive		Expensive
